# First report of *Echinococcus canadensis* (G7) in backyard pigs from the western highlands of Guatemala

**DOI:** 10.1017/S0031182025000150

**Published:** 2025-02

**Authors:** Roderico Hernández-Chea, Américo Yoel Aragón-Méndez, Alejandro Hun, Paola Morales-Ramírez, Ilde Silva, Federico Villatoro, Marion Wassermann

**Affiliations:** 1 Dirección Departamental de Redes Integradas de Servicios de Salud de Guatemala, Área Sur, Ministerio de Salud Pública y Asistencia Social, Amatitlán, Guatemala; 2 Facultad de Medicina Veterinaria y Zootecnia, Universidad de San Carlos de Guatemala, Guatemala, Guatemala; 3 Escuela de Estudios de Posgrado, Facultad de Medicina Veterinaria y Zootecnia, Universidad de San Carlos de Guatemala, Guatemala, Guatemala; 4 Instituto de Investigación en Ciencia Animal y Ecosalud, Facultad de Medicina Veterinaria y Zootecnia, Universidad de San Carlos de Guatemala, Guatemala, Guatemala; 5 Facultad de Biología Química y Farmacia, Universidad Galileo, Guatemala, Guatemala; 6 Facultad de Ciencias Jurídicas y Sociales, Universidad de San Carlos de Guatemala, Guatemala, Guatemala; 7 Institute of Biology, Department of Parasitology, University of Hohenheim, Stuttgart, Germany

**Keywords:** backyard pigs, cestode infections, echinococcosis, *Echinococcus canadensis* G7, Guatemala, haplotypes, *Taenia hydatigena*

## Abstract

*Echinococcus granulosus sensu lato* is the causative agent of cystic echinococcosis (CE), a globally distributed zoonotic infection. In Guatemala, no new data have been reported for the past 80 years on CE. To address this gap, a cross-sectional study at the municipal slaughterhouse of Quetzaltenango was conducted from March to August 2022 to determine the presence of *Echinococcus* sp. in backyard pigs. Moreover, the species and haplotypes, fertility status of hydatid cysts, association of fertility of the cysts to the sex of the pig and the size of cysts were investigated. For this purpose, 117 pigs were examined post-mortem, and cysts were extracted from their organs. Species identification was performed using nested polymerase chain reaction targeting the *cox1* gene, and a haplotype network was constructed. Generalized linear models (GLMs) were applied to assess correlation between cysts fertility, sex of the pig and diameter of the cyst. The study revealed a high prevalence of 38·46% of CE, and a minimum prevalence of *Taenia hydatigena* of 4·27%. Genetic characterization confirmed the presence of *Echinococcus canadensis* of the G7 haplogroup. Eight haplotypes unique to Guatemala were identified, along with one of global occurrence. Cysts from male pigs were 3·6 times more likely to be fertile than those from female pigs. A quadratic GLM determined that cysts with a diameter range of 2·09–4·20 cm had a higher probability of being fertile. The high prevalence of CE and the diversity of Guatemalan haplotypes confirm the endemicity of *E. canadensis* in this region.

## Graphical abstract

**Figure:**
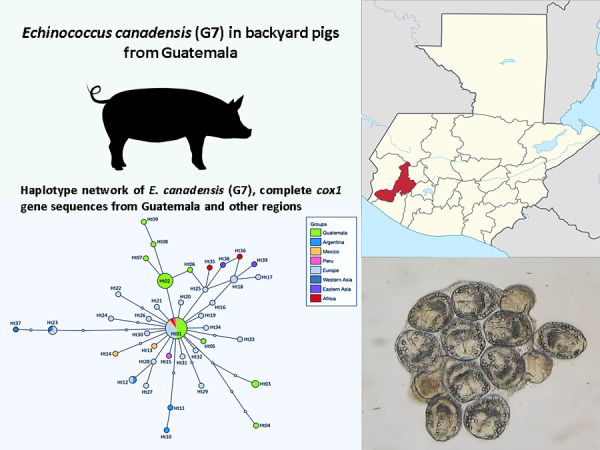

## Introduction

Cystic echinococcosis (CE) is a zoonotic disease caused by the larval stage of the medically important species complex *Echinococcus granulosus sensu lato* (*s.l.*). Human CE represents a significant public health concern with a worldwide distribution, being reported on all continents except Antarctica (Eckert et al., 2001). The life cycle of this parasite requires 2 mammalian hosts. Wild and domestic carnivores, particularly canids, act as definitive hosts and harbour the adult worms. Domestic or wild ungulates are the intermediate hosts and acquire the infection through the ingestion of the eggs. Once ingested, the oncospheres within the eggs hatch and migrate to internal organs, primarily the liver and lungs, where they develop into metacestodes (the larval stage), ultimately leading to the formation of hydatid cysts and cause CE. The life cycle is completed when the definitive hosts consume the infected organs of intermediate hosts (Eckert et al., 2001; Cardona and Carmena, 2013).

As a neglected tropical disease (NTD), CE is most prevalent in impoverished rural communities where animal husbandry is common. The worldwide disability-adjusted life years of CE has been estimated at 5 935 463 (Noguera et al., 2022). Cystic echinococcosis also negatively affects global livestock production, and the financial losses have been estimated to reach up to US$2 billion annually (Budke et al., 2006). Certain geographical regions face a particular severe impact from CE, posing a serious public health threat and causing substantial animal production losses. These include rural and grazing areas of South America (Moro and Schantz, 2006), the Mediterranean basin (Dakkak, 2010), North and East Africa (Romig et al., 2011),Western and Central Asia (Jenkins, 2005; Wang et al., 2008) and Oceania, particularly mainland Australia (Jenkins, 2006). Given its global significance and impact on public health, the WHO has included echinococcosis as one of the NTDs (World Health Organization, 2024). Based on morphological and particularly genetic differences, 5 species are currently recognized within the *E. granulosus s.l*. complex (Lymbery, 2017). These species are *Echinococcus granulosus sensu stricto* (*s.s*.), with its genotypes G1 and G3 (formerly the ‘sheep strain’ and ‘buffalo strain’, respectively); *Echinococcus equinus* (G4, also known as the ‘horse strain’); *E. ortleppi* (G5, ‘cattle strain’); the *E. canadensis* cluster, which consists of the genotype G6 and G7 (‘camel strain’ and ‘pig strain’), G8 (‘cervid strain’) and G10 (‘fennoscandian cervid strain’); and *E. felidis* (‘lion strain’) (Casulli et al., 2022). Within *E. granulosus s.l., E. granulosus s.s*. (G1) is the most important species in terms of public health, accounting for 88·5% of global CE infections in humans, and *E. canadensis* (G6 and G7) is the second most relevant species, responsible for 11% of human infections worldwide (Alvarez-Rojas et al., 2013). Only a few cases of *E. ortleppi* and, more recently, of *E. equinus* infections in humans have been reported, and no cases of *Echinococcus felidis* at all. Therefore, these species are of limited or no relevance to human health (Alvarez-Rojas et al., 2013; Kim et al., 2020; Macin et al., 2021).

Although the occurrence of *Echinococcus* spp. is well-documented across the American continent, both in North America and especially in South America (Deplazes et al., 2017; Romig et al., 2017), there is very limited information regarding this parasite in animal or human hosts from Central America. A few human cases have been reported from Nicaragua (Rausch and D´Alessandro, 2002), Costa Rica (probably acquired in Spain) (Brenes et al., 1977), Panama and Honduras (de Erazo and de Barahona, 1989; Sanchez et al., 1992; D’Alessandro and Rausch, 2008). Molecular identifications of the causative species were not conducted, but some cases could be attributed to neotropical echinococcosis caused by *E. vogeli* or *E. oligarthra* due to their morphological appearance. These 2 species are also implicated in the few documented infections in wild animals in Central America (Romig and Wassermann, 2024). Regarding CE in livestock, only 2 studies are known. One reports a single hydatid cyst in a pig from El Salvador (Llort, 1959), while the other, conducted nearly 80 years ago in Guatemala, reported that 1·71% (4870/284 772) of examined pigs were infected with CE (Aguilar, 1948), and 0·03% of the cattle harboured cysts (Pérez, 1949). This led to the assumption that *Echinococcus granulosus s.l.* is rare in most countries of Central America (Moro and Schantz, 2006). However, due to the limited data available, no definitive statement can be made in this regard.

To date, no molecular studies have been conducted to identify the species that cause CE in domestic animals in Central America. As a result, the prevalent *Echinococcus* species and their transmission dynamics in this region are unknown. This lack of information extends to the current status of *Echinococcus* spp. in Guatemala. However, the country exhibits social, economic, and epidemiologic characteristics, particularly in rural indigenous areas marked by extreme poverty and inadequate sanitation, that may facilitate the perpetuation of the life cycle of *Echinococcus* spp.

The suspected presence of *Echinococcus* spp. was confirmed through veterinary examinations of backyard pigs brought to the municipal slaughterhouse of the department of Quetzaltenango. These findings prompted further investigation into the prevalence, as well as number, size, and fertility status of cysts, and most importantly, the identification of the causative species of CE in the backyard pigs.

## Materials and methods

### Pilot study

In a pilot study on CE, 30 backyard pigs brought to the municipal slaughterhouse of the department of Quetzaltenango were examined in April 2019, for the presence of metacestodes of *Echinococcus* spp. Of these, 30% (9/30) were found to have hydatid cysts. These findings prompted further investigation into the prevalence, as well as number, size and fertility status of cysts, and most importantly, the identification of the causative species of CE in the backyard pigs brought to the municipal slaughterhouse of Quetzaltenango.

### Study area

A cross-sectional study was carried out to collect *Echinococcus* cysts from pigs between March and August 2022 at the slaughterhouse in the municipality of Quetzaltenango, located at longitude 14° 50ʹ 21″ N and latitude 91° 30ʹ 10″ W, at an altitude of 2331 m above sea level. The municipality of Quetzaltenango is situated in the western highlands of Guatemala and experiences rainy and dry seasons. The rainy season lasts from May to mid-November, with an annual rainfall of 3124 mm. The dry season, from December to May, is often characterized by little to no rainfall. The climate is classified as temperate, with average temperatures of 24 °C during the day and 5 °C at night (INSIVUMEH, 2024). Pig husbandry in the region is practiced in both free range and communal grazing systems, as well as in intensive pig farming. Quetzaltenango is the second largest city in Guatemala and its municipality represents the second most economically important in the country. The department has a population of approximately 799 101 inhabitants, with the municipality of Quetzaltenango home to around 180 706 inhabitants (Instituto Nacional de Estadística, 2018).

### Sample size

The sample size was determined using the formula *n* = *Z*^2^ *pq*/*e*^2^ (Martin et al., 1987), where *n* is the required number of individuals to be examined, *Z* = 1·96 is the standard normal 95th percentile, *p* is the estimated or known prevalence = 0·30 (based on the pilot study); *q* = 1 − *p*; and *e* is the precision of the estimate = 0·085, representing the allowable error of estimation. In this study, a confidence level of 95% was chosen; therefore, the minimum required sample size was 112 pigs. All male and female free range/backyard pigs ≥6 months of age brought to the municipal slaughterhouse of Quetzaltenango during the study period were included.

### Sample collection and parasitological examination

A copy of the project proposal and a letter requesting permission were sent to the slaughterhouse administrator and the official veterinarian. Following acceptance and approval, the research team provided the operators with a summary description of the project and the activities involved in carrying out the research within the slaughterhouse. The research team did not participate in any aspect of the slaughtering process. For each examined pig, the following data were recorded: sex and geographic origin (department, municipality and village). Due to the slaughterhouse routine, a post-mortem dental examination to determine the age of the animals was unfortunately not possible. With certainty it can only be stated that all animals were ≥6 months old. Based on their size, the majority were estimated to be no older than 12 months. Post-mortem, a detailed inspection of the organs (lungs and liver) of each pig was conducted by qualified veterinarians. When cysts of *Echinococcus* spp. or *Taenia* spp. were identified, the number and location of each cyst within the examined organs were recorded. Cysts were carefully extracted from each organ, stored in saline solution (0·9%) at 4 °C, and subsequently transported to the laboratory. The host tissues were carefully removed from the cysts using basic surgical instruments. The cysts were then cleaned by rinsing 5 times with 0·9% saline solution, and their dimensions (width and length) were measured to calculate the mean diameter (√ ((*r*_length_^2^ + *r*_width_^2^)/2)). The vesicular fluid of metacestodes was aseptically aspirated with a syringe, transferred to a flask, centrifuged at 7870·7 RCF for 10 min, and the supernatant was discarded. An aliquot was preserved, while another was deposited on a slide for the examination of wet smear under the microscope to detect protoscoleces of *Echinococcus* spp. The cysts were classified as fertile or sterile based on the presence or absence of protoscoleces. Inactive cysts were morphologically classified as calcified and/or caseous. A total of 32 hydatid cysts, characterized by diameter (cm) and/or the presence of protoscoleces, were collected from a proportional number of female and male pigs. These cysts were preserved in 70% ethanol (EtOH) and stored at −70 °C for molecular analysis in order to determine the species and genotypes of *Echinococcus*. The cysticerci of *T. hydatigena* were morphologically identified based on the arrangement of the rostellar hooks (Hobbs et al., 1990). For molecular species confirmation, two *T. hydatigena* cysticerci were also preserved in 70% EtOH.

### Preparation of samples for molecular analysis

DNA was prepared using the 0·02 M NaOH method as described previously (Nakao et al., 2003). Specifically, cyst fluid was examined microscopically, and a single protoscolex was transferred in a volume of 1 μL via pipette into 10 μL of 0·02 M NaOH solution. If protoscoleces were absent, a small piece (0·5 × 0·5 mm) of the germinal layer was instead transferred into 20 µL 0·02 M NaOH. The NaOH solution containing the parasite material was heated to 95 °C for 15 min. The same protocol was applied for *Taenia* sp. using a piece of the cysticercus. The resulting lysate was used directly as a DNA template for the polymerase chain reaction (PCR).

### Species identification by PCR and sequencing

For species identification, the complete mitochondrial cytochrome c oxidase subunit 1 (*cox1*) gene was amplified and sequenced. Nested PCR was performed using primers described previously (Hüttner et al., 2008; Wassermann et al., 2015). For the first PCR a 25 μL reaction was prepared containing 10 pmol each of the forward and reverse outer primers (forward primer: 5′ GTG GAG TTA CTG CTA ATA ATT TTG 3′ and reverse primer: 5′ TAC GAC TYA CTT ATC AC 3′), 10 mM Tris-HCl (pH 8·3), 50 mM KCl, 2 mM MgCl_2_, 200 μM of each dNTP, 0·625 U Ampli-Taq polymerase (Applied Biosystems), and 2 μL of the lysate. The 1975 bp long amplicons produced by the first PCR were used as DNA templates for the nested PCR, where the primers produced a 1842 bp fragment (nested forward primer: 5′ TTA CTG CTA ATA ATT TTG TGT CAT 3′ and nested reverse primer: 5′ GCA TGA TGC AAA AGG CAA ATA AAC 3′). Reaction mixture was set up to 50 μL using 20 pmol of each nested primer, 10 mM Tris-HCl (pH 8·3), 50 mM KCl, 2 mM MgCl_2_, 200 μM of each dNTP, 1·25 U Taq polymerase and 2 μL of the first PCR amplicons. Amplification conditions were identical for both PCRs: an initial denaturation step at 95 °C for 5 min, followed by 35 cycles of denaturation at 95 °C for 30 s, annealing at 55 °C for 30 s, and extension at 72 °C for 2 min. After a final extension step at 72 °C for 5 min, the PCR products were cooled to 4 °C. Nested PCR products were purified using the High Pure PCR product purification kit (Roche Diagnostics GmbH, Germany) according to the manufacturer’s instructions and sent to Microsynth Seqlab GmbH (Göttingen, Germany) for sequencing. Obtained DNA sequences were analysed and edited with GENtle v. 1.9 (Manske M. 2003, University of Cologne, Germany) and compared against existing sequences in the GenBank databases using the BLAST algorithm (www.blast.ncbi.nlm.nih.gov/blast.cgi).

### Haplotype network construction

The estimation of the gene genealogy and the construction of the haplotypes network were performed with the TCS v1.23 software with a connection limit of 95% (Clement et al., 2000). The resulting network was visualized with the online tool tcsBU (Santos et al., 2016). For comparison, *E. canadensis* sequences corresponding to the genotype G7 were retrieved from GenBank and included in the analyses. Each haplotype was included in the calculation once per country of occurrence (Table 5). Nucleotide and haplotype diversity indices for the Guatemalan samples were calculated using the DnaSP v. 6.12 software (Rozas et al., 2017). In addition, a phylogenetic analysis was performed using the maximum likelihood method with the HKY + I substitution model and bootstrap values calculated from 1000 replications, incorporating all haplotypes used in the haplotype analysis. This analysis was conducted using MEGA software.

### Statistical analysis

Qualitative variables are reported as frequencies and proportions. Prevalence values are reported as the percentage of pigs positive for cysts of *Echinococcus* sp. and *Taenia* sp., as accompanied by a 95% confidence interval (CI). Quantitative variables are reported as median, and interquartile ranges were obtained. The data collected during the visits to the slaughterhouse (sex, presence of cysts, fertile/sterile cysts, and diameter of the cysts) were recorded in Microsoft Excel 2007 and analysed using R Project for Statistical Computing v.4.1.1 (R Core Team, 2024). To assess a significant difference between the number of hydatid cysts with regard to the sex of the pigs, a generalized linear model (GLM) was constructed (without the animal identifier as a random factor) using a Poisson distribution for the variable number of cysts (95% CI).The diameter of the cyst, along with other variables influencing the descriptive analysis (measures of central tendency, graphics and measures of dispersion), were selected to fit a logistic regression model, as a GLM. The GLMs were fitted, including those variables, considering the pig (based on the animal identifier) as a random effect, using a binomial distribution for the response variable to calculate the adjusted odds ratio (OR). In addition, a quadratic version of the model was tested in order to explore for a unimodal correlation between the cyst’s fertility (response variable) and diameter of the cyst (predictor). *p* Values <0·05 were considered significant.

## Results

### Prevalence

**Figure 1.**
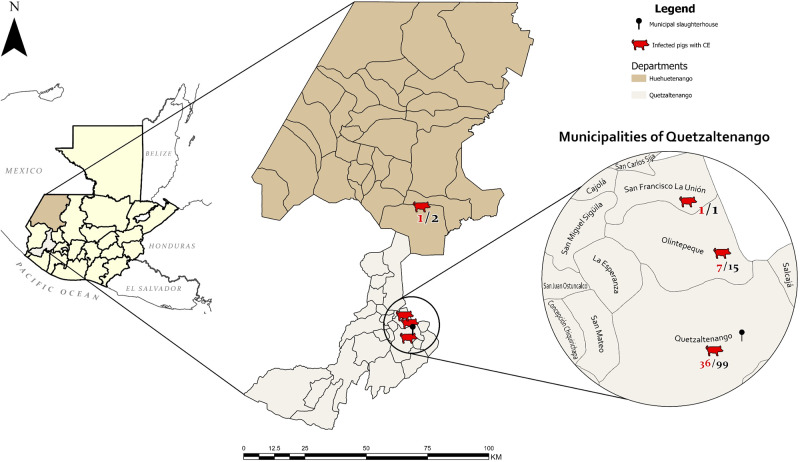
Map of the study area, department of Quetzaltenango, Guatemala, and the geographical location of the municipal slaughterhouse. Geographical origin of examined pigs, according to the data collected at the municipal slaughterhouse of Quetzaltenango. In red: number of CE-positive pigs, in black: total number of examined animals.

In this study, the livers and lungs of 117 backyard pigs were examined. Among these, 53% (62/117) were female and 47% (55/117) were male. Cestode infections were identified in 40·1% (47/117) of the examined pigs. Cystic echinococcosis was detected in 45 pigs, corresponding to a prevalence of 38·46% (45/117, 95% CI: 29·64%–47·28%). A higher proportion of male pigs 43·6% (24/55) were infected compared to females 33·9% (21/62), although this difference was not statistically significant (*p* = 0·5). *Taenia hydatigena* was detected in 5 livers. However, as only liver and lungs were examined in the present study, the true prevalence of *T. hydatigena* cannot be determined. Therefore, the minimum prevalence must be 4·27% (5/117, 95% CI: 0·61%–7·94%). The number and proportion of pigs infected with CE and *T. hydatigena* are shown in Table 1. The geographical location of pigs infected with CE is depicted in Figure 1.

**Table 1.** Number and percentage of backyard pigs tested positive and negative for cystic echinococcosis (CE) and *T. hydatigena* cysticercosis

Cestode infection	No. of backyard pigs (%)
CE	42 (35·90)
*T. hydatigena* cysticercosis	2 (1·71)
Coinfection CE/*T. hydatigena* cysticercosis	3 (2·56)
Negative	70 (59·83)
Total	117 (100)

### Parasitological examination and fertility of the cysts

**Figure 2.**
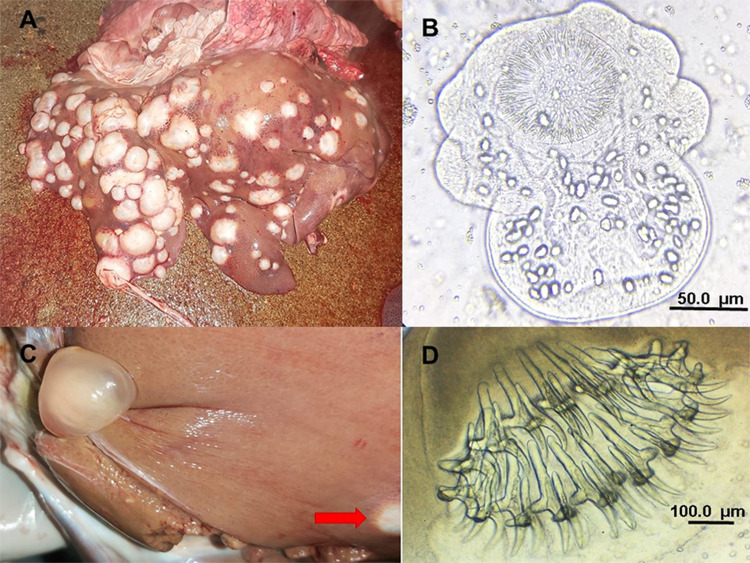
(A) Massive infection of CE found in the liver of a backyard pig; (B) evaginated protoscolex of *Echinococcus* extracted from vesicular fluid of 1 fertile hydatid cyst; (C) coinfection with *Taenia hydatigena* cysticercus and 1 hydatid cyst (red arrow) found in a liver; (D) rostellum of *T. hydatigena* with large and small rostellar hooks.

A total of 1140 cysts of parasitic origin were extracted from the organs of the examined backyard pigs. Of these, only 2 cysts were found in the lungs. The number of hydatid cysts per positive animal ranged from 1 to 563, with an average of 25·2 cysts per infected pig. Notably, 2 male pigs were heavily infected with 563 and 350 cysts, respectively. When these 2 individuals are excluded, the average number of cysts per infected animal decreases to 5·1. Discounting the heavily infected 2 pigs, male infected animals still exhibited on average more hydatid cysts (6·1) than females (4·1). In addition, 6 cysticerci of *T. hydatigena* were identified in 5 livers. The number and fertility status of hydatid cysts in the infected organs are shown in Table 2. Examples of hydatid cysts and cysticerci recovered from livers are illustrated in Figure 2. The numbers of extracted cysts from male and female pigs, with classification as fertile/sterile are presented in Table 3. Even after excluding the 2 male pigs with exceptionally high parasitic loads, male pigs had a greater total number of cysts and fertile cysts than females. Specifically, in 22 male pigs, 39 fertile cysts and 90 sterile cysts were observed.

**Table 2.** Number of fertile and infertile hydatid cysts, found in livers and lungs of the infected backyard pigs

		Hydatid cysts					
			Infertile cyst (%)				
Organ	No. of positive organs (%)	Fertile cysts (%)	Sterile	Calcified	Caseous	Fertility not determined	Total
Liver	45 (95·74)	221 (19·49)	851 (75·04)	9 (0·79)	19 (1·68)	32 (2·82)	1132 (99·82)
Lung	2 (4·26)	1 (0·09)	1 (0·09)	0	0	0	2 (0·18)
Total	47 (100·00)	222 (19·58)	852 (75·13)	9 (0·79)	19 (1·68)	32 (2·82)	1134 (100·00)

**Table 3.** Number and prevalence of infected pigs by sex and number and percentage of hydatid cysts with known fertility status

Sex of the host (total no. examined)	No. of positive pigs (%)	Total no. of cysts	Fertile cysts (%)	Sterile cysts (%)
Male (55)	24 (43·63)	1042	215 (20·63)	827 (79·37)
Male excl. 2^a^ (53)	22 (41·51)	129	39 (30·23)	90 (69·77)
Female (62)	21 (33·87)	60	7 (11·67)	53 (88·33)

a Two heavily infected male pigs exclude.

### Descriptive and multivariate analysis

Hydatid cysts with documented fertility status and measured diameters were included in the multivariate analysis. This analysis was performed on l92 cysts, randomly selected and extracted from 39 pigs. The mean diameter of the examined hydatid cysts was 3·0 cm, with a median of 1·52 cm, and an interquartile range of 1·30 cm. The mean and median values of the diameter of fertile and sterile cysts observed in the examined pigs are presented in Table 4.

**Table 4.** Mean, median and interquartile range values of the diameter of the examined hydatid cysts

Diameter	Mean (cm)	Median (cm)	Interquartile range
Total no. of cysts	3·0	1·52	1·30
Fertile cysts	2·23	2·06	0·75
Sterile cysts	1·57	1·25	1·30

**Figure 3.**
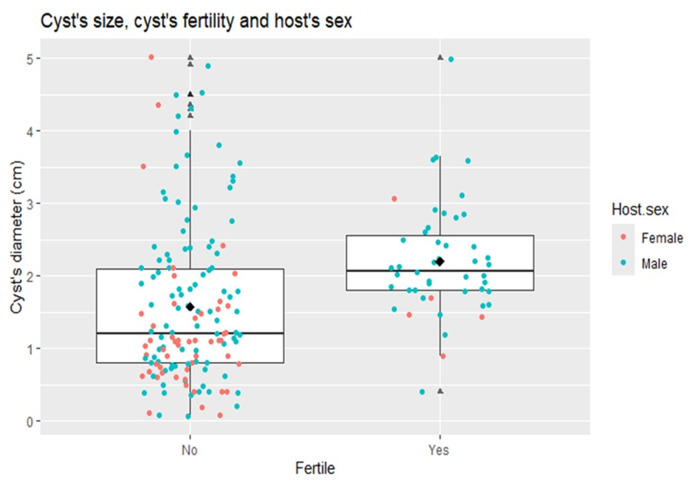
Boxplot of the size of fertile and sterile hydatid cysts in relation to the sex of the host. The horizontal line inside the box is the median. The median diameter of the sterile cysts is smaller than that of the fertile cysts; this explains why the box (which represents the distribution of the data according to the interquartile ranges) is higher. In the box the majority of fertile cysts correspond to males, showing the probability of association between the sex of the host and fertility, in this study.

The results of the GLM to assess the number of hydatid cysts with regard to the sex of the pigs indicated a significant difference (*p* = 0·01); and the ratio of females to males was 1:12 cysts (95% CI: 10·2–15·6). A GLM was constructed to evaluate the predictor variables related to fertility of hydatid cysts. Two variables were identified to be good predictors of hydatid cyst fertility (*p* = 0·01): the sex of the pig and the size of the cyst. For this purpose, 192 hydatid cysts (136 cysts from male pigs and 56 from female pigs) were included, as they had complete data on cyst diameter and fertility classification (sterile/fertile). The random effect of the host individual was justified, given the fact that the basal AIC (Akaike information criterion, null model) with the random effect included was lower (*p* < 0·001) than without this term. This suggests that factors inherent to the individual (e.g. sex of the animal) are associated with the probability of cyst fertility (Figure 3). The adjusted OR indicated that cysts from male pigs are 3·6 (95% CI: 1·3–9·8) times more likely to be fertile than those from female pigs. The probability of encountering more fertile cysts in male pigs was statistically significant (*p* = 0·005). After including the animal identifier as a random effect, only the cyst size (rather than sex of the pig) remained a suitable candidate to explain the probability of fertility of the cysts (*p* = 0·047). The difference in the medians of sterile and fertile cysts can be observed in Figure 3, illustrating a tendency for fertility to increase with diameter of the hydatid cysts. A quadratic GLM was fitted to the data in order to test the unimodal relationship between the variables cyst fertility and cyst size. According to this model, the highest probability for cyst fertility was in the size range of 2·09–4·2 cm in this age group of pigs (≥6 months).

### Identification of species and haplotype analyses

Thirty-two hydatid cysts (31 from the liver and 1 from the lung) collected from 21 pigs, along with 2 *T. hydatigena* isolates from 2 individual pigs, were subjected to molecular analyses to identify or confirm the respective species. Partial sequencing of the *cox1* gene from the putative *T. hydatigena* samples and subsequent comparison with GenBank entries, confirmed their identity. All hydatid cysts were caused by *Echinococcus canadensis* of the G7 haplogroup. The complete *cox1* gene sequence could be obtained from 28 cyst samples from 20 pigs, only a partial *cox1* sequence from 4 cysts. Twenty-two cysts from 16 pigs, with a maximum of 2 cysts analysed per animal, originated from the municipality Quetzaltenango, 5 cysts from 3 pigs from Olintepeque, and the single isolate from San Francisco La Unión.

### Haplotype analyses

**Figure 4.**
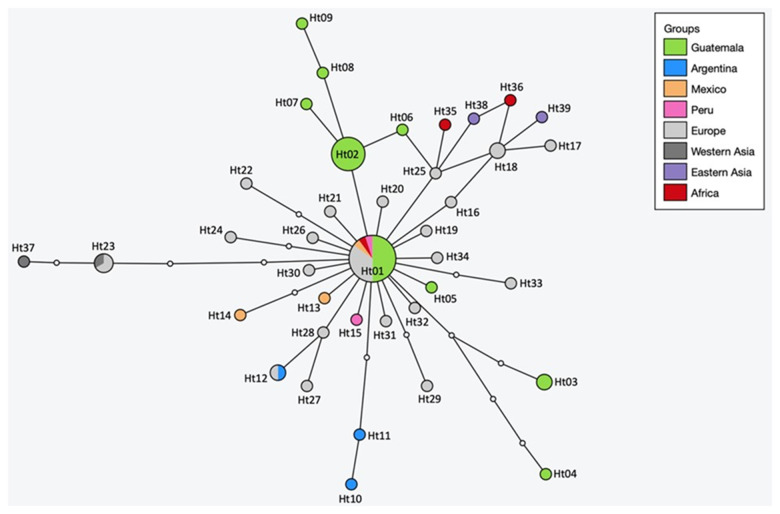
Haplotype network of *Echinococcus canadensis* G7 *cox1* gene sequences (1608 bp) from Guatemala and other countries. The size of the circles indicates the frequency of haplotypes and small white dots showing hypothetical haplotypes (nucleotide exchanges).

Nine haplotypes were identified among the 28 complete *cox1* sequences of the *E. canadensis* isolates, which were submitted to the NCBI GenBank under the Accession Numbers PP716595–PP716603. The analyses of the haplotype (H_d_) and nucleotide diversity (N_d_) of the Guatemalan samples resulted in H_d_: 0·759 ± 0·057 and N_d_: 0·00101 ± 0·00022. A haplotype network was constructed with the complete *cox1* sequences of all Guatemalan isolates (comprising 9 haplotypes) and *E. canadensis* G7 entries available in GenBank (Figure 4, Table 5). The haplotypes were included in the calculation once per country of origin, except for those from Guatemalan, where all 28 sequences were incorporated to display the frequency of the respective haplotypes found within the country.

**Table 5.** Geographic origin, haplotype, accession numbers and references of *Echinococcus canadensis* G7 sequences used for the network analysis

Geographic region	Country	Haplotype	Accession number	Reference
America	Guatemala	Ht01	PP716595	This study
		Ht02	PP716596	”
		Ht03	PP716597	”
		Ht04	PP716598	”
		Ht05	PP716599	”
		Ht06	PP716600	”
		Ht07	PP716601	”
		Ht08	PP716602	”
		Ht09	PP716603	”
	Argentina	Ht10	MH300955	Laurimäe et al. (2017)
		Ht11	MH300962	”
		Ht12	MH300968	”
	Mexico	Ht01	MH300972	”
		Ht13	MH300980	”
		Ht14	MH300981	”
	Peru	Ht01	AB777924	Nakao et al. (2013)
		Ht15	AB777925	”
Europe	France	Ht16	KX010856	Addy et al. (2017)
		Ht17	KX010857	”
		Ht01	MH300986	Laurimäe et al. (2017)
		Ht18	MH301008	”
	Serbia	Ht19	KX510133	Addy et al. (2017)
		Ht20	KX010863	”
		Ht21	KX010864	”
		Ht22	KX010865	”
		Ht23	KX010866	”
		Ht24	KX010867	”
		Ht25	KX010868	”
		Ht01	KX010869	”
		Ht26	MH300984	Laurimäe et al. (2017)
	Slovakia	Ht01	KX010869	Addy et al. (2017)
		Ht27	KX010858	”
		Ht28	KX010859	”
	Poland	Ht29	MH301003	Laurimäe et al. (2017)
		Ht01	MH301004	”
		Ht30	MH301006	”
		Ht12	AB235847	Nakao et al. (2007)
	Romania	Ht31	MH300982	Laurimäe et al. (2017)
		Ht01	MH300983	”
	Hungary	Ht32	KX010860	Addy et al. (2017)
		Ht33	KX010861	”
		Ht01	KX010869	”
	Italy	Ht18	MH301018	Laurimäe et al. (2017)
	Spain	Ht01	MH300985	”
	Ukraine	Ht23	MH301021	”
	Lithuania	Ht34	MH301020	”
Africa	Sudan	Ht35	KX010846	Addy et al. (2017)
	Kenya	Ht01	KX010869	”
	Namibia	Ht36	KX010854	”
Western Asia	Armenia	Ht23	KX010866	”
		Ht37	KX231667	”
Eastern Asia	Mongolia	Ht38	AB893263	Ito et al. (2014)
		Ht39	MH300971	Laurimäe et al. (2017)

The global network has a star-shape structure, with the main haplotype (Ht01) having a worldwide distribution. Ht01 was detected in 7 European countries, as well as in Kenya, Peru, Mexico and in 10 samples from Guatemala. The other haplotypes are arranged around Ht01, differing by 1–5 nucleotides. Of the 39 haplotypes, only 3 could be found in more than 1 geographic region. Besides Ht01, Ht12 was detected in Argentina and Poland and Ht23 in Europe (Ukraine and Serbia) and in Western Asia (Armenia). Ht18 was also detected in 2 countries, but within the same geographical region, France and Italy. It is noteworthy that the Guatemalan Ht02 was found as frequent as Ht01 in the country. Both occurred 10 times, making them equally represented in Guatemala. The Guatemalan haplotype Ht03 was detected twice, the remaining 6 once each. The distance between the 2 most distantly related haplotypes in the global network (Ht04 and Ht37) is 9 nucleotide substitutions, and between the most distant Guatemalan (Ht04 and Ht09) 7 nucleotides.

All 9 Guatemalan haplotypes were identified in the 22 samples originating from the municipality of Quetzaltenango, whereas the 5 isolates collected from 3 pigs from Olintepeque, and the single isolate from San Francisco La Unión belonged to Ht01. Overall, 5 pigs were infected with Ht01, 7 with Ht02, 2 had a double infection with Ht01 and Ht02, 1 pig was infected with Ht03, another had a double infection with Ht03 and Ht05, 1 pig was infected with Ht04 and Ht07, and 3 pigs had single infections with Ht06, Ht08 and Ht09, respectively.

To investigate the relationships among *E. canadensis* G7 haplotypes and assess whether the Guatemalan haplotypes might occupy a phylogenetically ancestral position, a phylogenetic tree was constructed using maximum likelihood analysis (Supplementary Figure S1). The tree indicates that all haplotypes, except Ht25 and its connected haplotypes (African Ht35, Ht36, Eastern Asian Ht38 and European Ht17 and Ht18), evolved from Ht01, as also visualized in the haplotype network. These basal haplotypes belong to the G7b haplogroup as defined by Laurimäe et al., 2017. Haplotype Ht39 (referred to as Gmon in Gonçalves Baptista et al., 2023) is most closely related to G6 and is most basally positioned within the G7 cluster. However, the bootstrap values for all nodes are extremely low, suggesting that the topology may change significantly with a larger dataset (Supplementary Figure S1).

## Discussion

Free-range or backyard pig farming is a common practice in Guatemala, and it is estimated that more than 69% of pig farming relies on backyard rearing in the country (MAGA, 2021; INE, 2021). In rural areas where backyard pig farming predominates, home slaughter is a common practice, and pork is consumed without prior veterinary inspection. Two types of pig population are brought to the municipal slaughterhouse of Quetzaltenango: pigs from intensive production systems and animals from backyard rearing. This cross-sectional study focused on the backyard pig population; building on findings from a pilot study in which 9/30 backyard pigs were detected positive, indicating a considerable problem with echinococcosis in this population. Furthermore, these pigs play a crucial role in sustaining the life cycle due to the practice of home slaughtering. A review of veterinary inspection data from the past 5 years at the abattoir also showed that a greater proportion of offal was condemned from backyard pigs than from intensively reared pigs. The last report on *Echinococcus* spp. in the country was carried out in a municipal slaughterhouse located in Guatemala City (Aguilar, 1948). During the period 1936–1942, the livers of 284 772 pigs were examined and 1·71% (4870 individuals) were found to be infected with *Echinococcus* sp. This low prevalence contrasts sharply with the results of the present study, where 38·46% (45/117) of the examined pigs were tested positive for *Echinococcus* sp. One possible explanation for this considerable difference could be that the study of Aguilar (1948) included pigs from all farming systems, both intensive and backyard. In contrast, the current study focused exclusively on backyard. Further studies are needed to estimate a national prevalence of *Echinococcus* spp. in pigs, including both backyard/free range and intensive reared populations.

The high prevalence of *Echinococcus* sp. related to backyard pig rearing has also been reported in studies from, e.g. Lithuania or Austria (Bružinskaitė et al., 2009; Schneider et al., 2010). This is, of course, associated with home slaughtering of pigs and the presence of free-roaming dogs, which are either intentionally fed with offal deemed unsuitable for human consumption or have access to the discarded offal (Schneider et al., 2010). Regarding the geographical origin of the pigs infected with *E. canadensis*, it could be shown that the infection is more widespread and not confined to the municipality of Quetzaltenango. *Echinococcus canadensis* was detected in pigs from the municipalities of San Juan Olintepeque and San Francisco la Unión (both within Quetzaltenango), and from the neighboring department Huehuetenango to the north. These results indicate that the conditions required to sustain the life cycle of *E. canadensis* are given in many regions of the western highlands of Guatemala. Consequently, further studies are necessary to identify endemic areas of *E. canadensis* and *E. granulosus* (*s.l.*) within domestic ungulate species in Guatemala.

Regarding the organ location of CE, of the 1134 cysts examined in this study, 99·82% (1132) were located in the liver, and 0·18% (2) in the lung. These results are consistent with other studies reporting that the liver is the most frequent infected organ with *Echinococcus* sp. in swine, followed by the lungs (Bružinskaitė et al., 2009; Umhang et al., 2014; Sierra Ramos and Valderrama-Pomé, 2017; Sgroi et al., 2019). Regarding the fertility of *E. granulosus s.s*. cysts, reports from domestic pigs in Europe, Asia, and South America indicate fertility rates ranging from 0% to 100% (Sánchez et al., 2012; Cardona and Carmena, 2013; Tigre et al., 2015). In this study, the fertility rate of the cysts was relatively high at 20·15% (222/1102 cysts), considering the relatively young age of the animals, and that fertility increases with advancing age of the animals or cysts, respectively (Bružinskaitė et al., 2009; Sierra Ramos and Valderrama-Pomé, 2017; Sgroi et al., 2019). Comparable rates of fertile cysts of *E. canadensis* G7 have been reported previously in domestic pigs, e.g. 23% in Lithuania (Bružinskaitė et al., 2009), 30% in Corsica (Umhang et al., 2014) and 31% in Cape Verde (Gonçalves Baptista et al., 2023). However, it can even reach 56·9% in wild boars in Italy (Sgroi et al., 2019), although the examined boars were generally older than commercially raised pigs. The rapid development of fertility in young cysts may represent an adaptive strategy of the parasite to its pig host, which generally does not reach an advanced age.

Interestingly, in this study, male pigs were more frequently infected, harboured on average more hydatid cysts than female pigs, and the cysts were more often fertile. The fertility of hydatid cysts was associated with the sex of the infected pigs, with a statistically significant higher probability of finding more fertile cysts in male than in female pigs (*p* = 0·005). Previous studies that differentiated between the sexes found no significant differences in the prevalence of infection in males and females (Lidetu and Hutchinson, 2007; Sierra Ramos and Valderrama-Pomé, 2017; Sgroi et al., 2019). However, these studies did not investigate potential differences between the sexes concerning the number of cysts and their fertility. In the 1 exception, where at least the fertility status of cysts in male and female pigs was examined, significantly higher fertility was found in female pigs (Sierra Ramos and Valderrama-Pomé, 2017). This is in contrast to our results. However, the authors did not provide information on possible age differences between the sexes at the time of slaughter, which renders it unclear whether the female animals were perhaps generally older, which could explain the higher fertility of their cysts. In the present study, the vast majority of animals were between 6 and 12 months old and belonged to the same age group. The results obtained in this study for male pigs, the higher number and increased fertility of hydatid cysts, are challenging to explain. One study found no statistical difference in the number and fertility of the cysts between the sexes in feral pigs (Lidetu and Hutchinson, 2007). In an experimental model, it was demonstrated using BALB/c mice that, after inoculation of protoscoleces, females presented a greater number of hydatid cysts in the liver than males, with a more pronounced granulomatous response observed in females. It was also observed that estradiol levels increased during chronic stages of the infection, while testosterone levels decrease (Blancas Mosqueda et al., 2007). However, the effect of these hormones and the granulomatous response on the fertility of the hydatid cysts was not investigated. According to a meta-analysis by Poulin (1996), male hosts are generally more susceptible to helminth infections than female hosts. Whether this applies to *E. granulosus s.l.* remains uncertain, as there are no studies addressing the natural transmission routes and intermediate hosts of the respective *Echinococcus* species.

In the current study, the cysts of *E. canadensis* with a diameter range of 2·09–4·20 cm had a higher probability of being fertile. There are no comparable studies, as a specific categorization of the cyst size (small, medium, and large) has not been implemented for *E. canadensis* in swine hosts. Therefore, further studies in slaughterhouses and at the community level are necessary to develop this classification specifically for this species in this host. Such a classification could be valuable in association with fertility rate, to understand factors related to the biological and epidemiological success of the parasite.

Twenty-eight CE cysts were subjected to molecular analyses of the complete mitochondrial *cox1* gene, revealing 9 genetic variants. The haplotype network, calculated with the Guatemalan and other *E. canadensis* G7 sequences from GenBank, showed that the most common haplotype worldwide (Ht01) is also present in Guatemala, detected in 10 CE specimens. This genetic variant has also been identified in European countries, Mexico, Peru and Africa and belongs to the haplogroup G7a, as defined by Laurimäe et al. (2017) The remaining 8 Guatemalan haplotypes were unique. All 9 Guatemalan haplotypes were identified in samples from the municipality of Quetzaltenango, whereas only Ht01 was detected in isolates from Olintepeque and San Francisco La Unión. However, this discrepancy may be attributed to the unequal number of isolates from different regions, with the majority originating from Quetzaltenango. Additionally, the geographic distance between the origins of pigs is approximately 20 km, suggesting that the observed differences are not likely due to geographic separation. Nonetheless, additional samples from Olintepeque and San Francisco La Unión would be needed to determine whether similar diversity in haplotypes can be found in these regions. The haplotype Ht02 was detected in 9 pigs, while Ht01 was found in 7 pigs, 2 of which had a double infection with both haplotypes. Given the close numbers, this suggests that both haplotypes are similarly established in the region. Additionally, 2 more pigs, out of the 8 in which 2 cysts were analysed, exhibited double infections with different haplotypes. This suggests a high infection pressure, highlighting the need for future studies to analyse multiple cysts per animal whenever possible to gain deeper insights into the local infection pressure.

From a global perspective, most of the 39 haplotypes analysed in this study were found exclusively in 1 geographical region. Including Ht01, only 3 haplotypes have been described from more than 1 region of the world. From a phylogenetic perspective, Ht01 appears to be the ancestor of all G7a haplotypes analysed in this study. However, this interpretation should be approached with caution due to the weak bootstrap values. Considering the network, the diversity of the Guatemalan samples appears to be relatively high. There are 7 nucleotide substitutions between the most distant Guatemalan haplotypes. Globally, the most distant gene variants are separated by only 2 additional nucleotides, with a total of 9 exchanges. Considering that the samples were collected from a region with a radius of approximately 10 km, they appear to have undergone substantial differentiation compared to the haplotypes found globally. In addition, the Guatemalan Ht02 occurs just as frequently in the country as Ht01. This could be an indication that this parasite has been endemic in Guatemala for a long time. The typical structure characterized by a central haplotype, from which all other haplotypes gradually diverge, which is often observed following a recent introduction, does not exist here (Nakao et al., 2010; Yanagida et al., 2012). However, it is also possible that Ht01 and Ht02 were introduced at the same time and the other haplotypes evolved from them. What challenges this hypothesis is the fact that Ht02 has not (yet) been detected elsewhere. Further studies and additional genetic data, particularly from Central and South America, would be needed to clarify this question.

The true prevalence of *T. hydatigena* could not be determined in this study due to the limitation of the investigation to liver and lungs. Larvae of *T. hydatigena* often migrate from the liver and develop to cysticerci in the peritoneal cavity, where they attach to the peritoneum, omentum, or mesentery, and are not primarily restricted to the liver (Eckert et al., 2001; Miller et al., 2012). Nevertheless, 5 pigs were found positive, resulting in a minimum prevalence of 4·27%. Similar low prevalence values have been reported from Tanzania with 1·4% (Ngowi et al., 2004), 6·7% in Ghana (Permin et al., 1999) and 6·5% in Cameroon (Assana et al., 2019). In contrast, other studies have revealed a higher prevalence of *T. hydatigena* cysticercosis in pigs, e.g. 18·0% in Vietnam (Nguyen et al., 2020) and 22·4% in Laos (Conlan et al., 2012). This parasitic infection in pigs, similar to echinococcosis, is associated with the traditional backyard farming, where pigs have access to fecal material from free-roaming dogs. These dogs are often fed condemned organs containing *T. hydatigena* cysticerci or *Echinococcus* spp. metacestodes (Nguyen et al., 2020). It was therefore unsurprising that this study not only identified both parasites but also documented co-infections in 3 cases (2·56%). Few studies have reported co-infection of these cestodes in swine. Monteiro et al. (2015) observed a low prevalence of *E. granulosus s.l*. compared to *T. hydatigena* in southern Brazil (10% and 57%, respectively), while the opposite result was found in wild boars from Tunisia (19% and 4%) (Lahmar et al., 2019). The occurrence of *E. canadensis* and *T. hydatigena* in the present study, suggests that there is a practice of feeding dogs with contaminated viscera from pigs, thereby perpetuating the life cycle of these cestodes.

*Echinococcus canadensis* is the second most important species causing human echinococcosis worldwide and is the primary causative agent of human cases in some countries of Central Europe like Austria and Poland (Schneider et al., 2010; Dybicz et al., 2013). More recently, human cases have also been reported in South America, specifically in Argentina (Debiaggi et al., 2017) and Brazil (Das Neves et al., 2024). Although human infections have not yet been reported in Guatemala, *E. canadensis* G7 is known to be pathogenic to humans and the present study demonstrates that this parasite is widespread in the western highlands of the country. Therefore, ultrasound surveys of the human population, particularly in rural areas, should be conducted to determine whether human CE cases truly do not exist or if they are simply underreported. Additionally, there is a pressing need to investigate the prevalence of *E. canadensis*, or *Echinococcus* in general, in the dog population to assess the risk of transmission of this parasite to the human population. Moreover, awareness campaigns should be implemented to discourage the practice of feeding raw offal to dogs.

The occurrence of *E. canadensis* and *T. hydatigena* in this study, suggests that there is a practice of feeding dogs with contaminated viscera of pigs with metacestodes; therefore, a domestic life cycle is well stablished. The role of other canids as definitive hosts of *Echinococcus* spp. in Guatemala is unknown. *Canis latrans* (coyote) could be the most suitable wild definitive host (Romig and Wassermann, 2014); however, they are very rare in the region and are therefore unlikely to play a significant role in sustaining the life cycle of *Echinococcus* spp. Furthermore, it would be important to know the role of other possible definitive hosts, such as *Urocyon cinereoargenteus* (grey fox).

## Supporting information

Hernández-Chea et al. supplementary material Hernández-Chea et al. supplementary material

## Data Availability

Data supporting results are provided within the article and available on GenBank under accession numbers PP716595-P716603.

## Supplementary material

The supplementary material for this article can be found at https://doi.org/10.1017/S0031182025000150.
