# Supplementary figures and images for: First report of Echinococcus canadensis (G7) in backyard pigs from the western highlands of Guatemala
Source: Parasitology. 2025 Feb 14;152(2):205–16. doi: 10.1017/S0031182025000150 (PMC12089453; doi:10.1017/S0031182025000150)

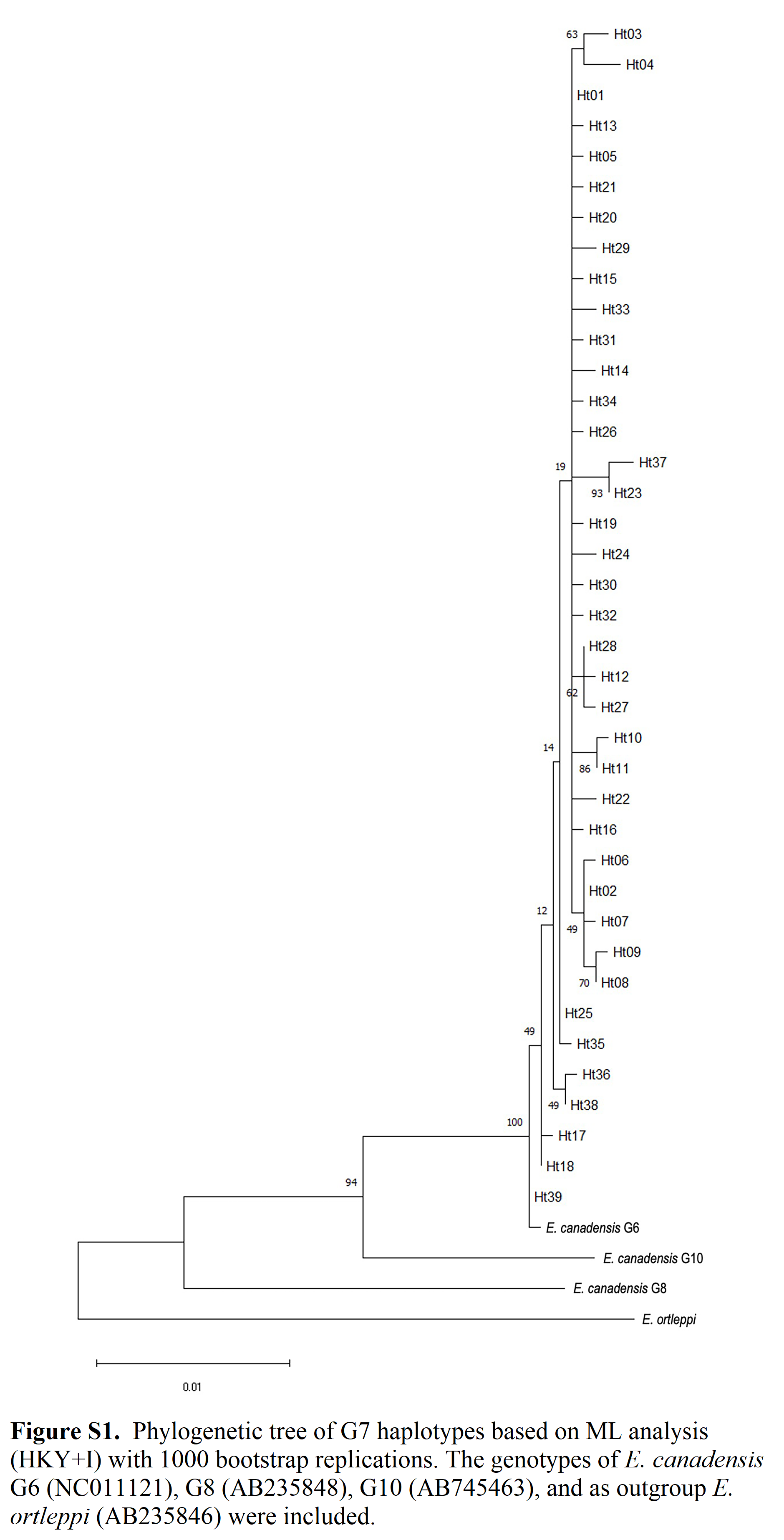

Supplement: Hernández-Chea et al. supplementary material [file S0031182025000150sup001.tif]
